# Dynamics of YAP localization and transcript activity in human oocytes and granulosa cells across early-stage folliculogenesis: an exploratory investigation

**DOI:** 10.1007/s10815-025-03668-2

**Published:** 2025-09-26

**Authors:** Jordan H. Machlin, Kate Potocsky, Robin E. Kruger, Jason Spence, Vasantha Padmanabhan, Ariella Shikanov

**Affiliations:** 1 Cellular and Molecular Biology Program, University of Michigan, Ann Arbor, MI 48109 USA; 2 School of Kinesiology, University of Michigan, Ann Arbor, MI 48109 USA; 3 Department of Biomedical Engineering, University of Michigan, Ann Arbor, MI 48109 USA; 4 Department of Internal Medicine, University of Michigan, Ann Arbor, MI 48109 USA; 5 Department of Cell and Developmental Biology, University of Michigan, Ann Arbor, MI 48109 USA; 6 Department of Obstetrics and Gynecology, University of Michigan, 2126 Lurie Biomedical Engineering, 1101 Beal Avenue, Ann Arbor, MI 48109 USA; 7 Department of Pediatrics and Communicable Diseases, University of Michigan, Ann Arbor, MI 48109 USA; 8 Department of Molecular and Integrative Physiology, Ann Arbor, MI 48109 USA

**Keywords:** Human ovarian cortex tissue, Follicle activation, YAP, CCN2

## Abstract

**Purpose:**

The transcription factor yes-associated protein (YAP) has been implicated in the regulation of murine follicle activation at the level of granulosa cells (GCs); however, less is known about its role in human folliculogenesis. This study aimed to explore the localization dynamics of YAP in the nucleus and cytoplasm of oocytes and granulosa cells in human ovarian follicles.

**Methods:**

YAP nuclear localization and mRNA expression of its gene target, connective tissue growth factor (CCN2), were investigated in oocytes and GCs across primordial, transitioning primordial, primary, and secondary follicles in human ovarian cortical tissue cultured up to 48 h. Slow-frozen and thawed human ovarian cortex squares (10 × 10 × 1 mm) were cut into smaller tissue strips (1 × 5 × 1 mm), and these strips were either fixed immediately (Timepoint 0) or cultured for 6, 24, or 48 h (*n* = 3 per timepoint). We analyzed oocytes and GCs from a total of 562 human follicles—112 primordial, 373 transitioning primordial, 65 primary, and 12 secondary.

**Results:**

Within each timepoint, YAP nuclear localization in GCs showed significantly greater abundance at the primary stages compared to the primordial stages but remained unchanged in oocytes. Furthermore, CCN2 mRNA expression in GCs increased significantly between the primordial and secondary stages of folliculogenesis, indicating YAP is transcriptionally active in granulosa cells of growing follicles.

**Conclusion:**

Our findings provide the important insight that YAP activity in granulosa cells could be a key regulator within human follicles transitioning from primordial to primary stages, a role that is likely conserved between humans and mice.

**Supplementary Information:**

The online version contains supplementary material available at 10.1007/s10815-025-03668-2.

## Introduction

Mechanotransduction is the process by which mechanical stimuli are translated into biochemical signals within cells, influencing cell behavior and gene expression [1]. The Hippo signaling cascade was identified as a mechanosensitive pathway, where transcriptional regulators of this pathway emerged as differentially regulated when mammary epithelial cells were grown on substrates with varying stiffness [2]. Activated Hippo signaling initiates a serine kinase cascade that phosphorylates and inactivates the downstream transcription factors yes-associated protein (YAP) and its transcriptional coactivator TAZ, which are then retained in the cytoplasm [3–5]. When Hippo signaling is inactive or disrupted, YAP and TAZ translocate to the nucleus, where they act with transcriptional enhanced associate domain (TEAD) to increase the expression of connective tissue growth factors (CCN2) and apoptosis inhibitors [4, 5]. Increased mechanical stress keeps Hippo signaling active and cell growth inhibited, while removing mechanical stress signals through physical disruption inhibits the pathway, allowing growth initiation [6, 7].

The structure of the human ovary is complex and mechanically responsive. Dormant, early-stage follicles reside in a stiff, collagen-rich outer cortex, and activated, later-stage follicles are found in the softer inner medulla [8]. An increasing body of evidence suggests that the Hippo signaling pathway is involved in regulating follicle activation in mice. Murine studies demonstrated that YAP knockdown leads to inhibition of follicle growth, with most follicles remaining in the primordial stage, while overexpression of YAP increases the population of growing follicles [9]. In addition, mechanical fragmentation of mouse ovaries increased F-actin polymerization and inhibited the Hippo pathway, resulting in an accumulation of YAP in the nucleus of granulosa cells (GCs) and an increase in CCN transcripts [5, 10, 11].

Whether the role of the Hippo pathway in follicle activation is conserved in human tissue remains the topic of investigation. This pathway’s sensitivity to mechanical disruption makes it a great candidate for understanding the rapid follicle activation observed following ovarian tissue fragmentation prior to cryopreservation for fertility preservation [11, 12]. Moreover, this pathway could be harnessed for use in alternative fertility preservation methods like in vitro activation (IVA), where follicles are activated and cultured to ultimately obtain mature fertilizable eggs [13, 14]. When looking into the Hippo pathway in human tissue, some studies noted increased nuclear expression of YAP in granulosa cells of primordial follicles after mechanical disruption and a subsequent increase in downstream YAP target transcripts, while others saw no change [15–19]. However, little is known about YAP dynamics in transitioning primordial follicles after activation or later stage follicles, including primary and secondary, in human ovarian tissue. Our study aimed to investigate YAP nuclear localization in human ovarian follicles immediately after thawing cryopreserved ovarian tissue and after short-term culture. We analyzed both oocytes and somatic cells across four distinct follicle stages, up to 48h after tissue fragmentation and culture. This work expands our understanding of the role of YAP during follicle activation, contributing to the broader goal of developing alternative fertility preservation methods.

## Methods

### Collection of human ovarian tissue

This study used ovarian tissue from two de-identified deceased donors procured through the International Institute for the Advancement of Medicine (IIAM) and the associated Organ Procurement Organization (OPO), which performed the tissue harvest. Both donors were pre-menopausal, and the provided medical records indicated no pathological conditions impacting ovarian function. The age, BMI, recorded “race,” cold ischemic time, and the ovarian dimensions are shown in Table 1. Cold ischemic time (CIT) was calculated as the interval between cross-clamp time of the donor (and subsequent cessation of arterial blood flow to the ovaries) in the operating room and the start time of tissue processing after arrival at the laboratory. Before cross-clamping, the organs were perfused with Belzer University of Wisconsin® Cold Storage Solution (Bridge of Life, SC, USA), Custodiol® HTK (Histidine-Tryptophan-Ketoglutarate) Solution (Essential Pharmaceuticals, NC, USA), or SPS-1 Static Preservation Solution (Organ Recover Systems, IL, USA).

**Table 1:** Donor demographics

Donor #	Age	BMI (kg/m^2^)	Race	Cold ischemic time (h)	Average ovary volume	Ovary 1	Ovary 2
						Dimensions (***L*** × ***W*** × ***H***) (cm)	Dimensions (***L*** × ***W*** × ***H***) (cm)
1	27	28.9	White	25.6	2	2.5 × 1.7 × 1	2.4 × 1.6 × 0.9
2	23	50.7	White	23.6	7	3.5 × 2.7 × 1.2	4.0 × 3.0 × 1.3

### Ethical approval process

The IIAM procures tissue and organs from Organ Procurement Organizations (OPOs) for non-clinical research [20]. OPOs comply with state Uniform Anatomical Gift Acts (UAGA) and are certified and regulated by the Centers for Medicare and Medicaid Services (CMS). The OPOs are members of the Organ Procurement and Transplantation Network (OPTN) and the United Network for Organ Sharing (UNOS) and operate under a set of standards established by the Association of Organ Procurement Organizations (AOPO) and UNOS. Informed, written consent from the deceased donor’s family was obtained for the tissue used in this publication. A biomaterial transfer agreement is in place between IIAM and the University of Michigan that allows the use of the tissue for pre-clinical research that does not involve the fertilization of gametes. The use of deceased donor ovarian tissue in this research is categorized as “not regulated,” per 45 CFR 46.102 and the “Common Rule,” and complies with the University of Michigan’s IRB requirements as such.

### Tissue processing

The tissue processing was done aseptically in a biosafety cabinet. After receiving donor tissues, the ovaries were separated from the uterus and fallopian tubes. The ~ 1-mm-thick ovarian cortex was removed using a custom cutting guide (Reprolife Japan, Tokyo) and cut into 10 × 10-mm squares. The “squares” were then aseptically transferred into holding media (Quinn’s Advantage Medium with HEPES (QAMH), 10% Quinn’s Advantage Serum Protein Substitute (SPS), CooperSurgical, Måløv, Denmark) before cryopreservation.

### Slow freezing procedure

The methods described by Xu et al. were used for slow freezing [21]. Briefly, squares of cortical tissue approximately 10 mm × 10 mm × 1 mm were placed into cryovials (Nunc, Roskilde, Denmark) filled with pre-cooled cryoprotectant media (QAMH, 10% SPS, 0.75 M dimethyl sulfoxide (DMSO) (Sigma Aldrich, St. Louis, USA), 0.75 M ethylene glycol (Sigma Aldrich, St. Louis, USA), 0.1 M sucrose (Sigma Aldrich, St. Louis, USA)) and equilibrated at 4 °C for at least 30 min. After equilibration, cryovials were loaded into the Cryologic Freeze Control System (Cryologic, Victoria, Australia). Vials were then frozen via the following protocol: (1) cooled from 4 to − 9 °C at a rate of − 2 °C/min, (2) equilibrated for 6 min at − 9 °C, (3) seeded manually using large swabs cooled by submersion in liquid nitrogen, (4) held for 4 min at − 9 °C, (5) cooled to − 40 °C at a rate of − 0.3 °C/min, and (6) plunged into liquid nitrogen and stored in a cryogenic storage dewar until thawed for use.

### Tissue thawing procedure

Vials with ovarian tissues were removed from liquid nitrogen and placed in a 37 °C bath. Once the cryoprotectant media in the vial had thawed, the tissue was removed from the vial and put into Thaw Solution One (0.5 M DMSO, 0.5 M ethylene glycol, 0.1 M sucrose, 10% SPS in QAMH) for 10 min. Tissue was then incubated sequentially in Thaw Solution Two (0.25 M DMSO, 0.25 M ethylene glycol, 0.1 M sucrose, 10% SPS in QAMH), Three (0.1 M sucrose, 10% SPS in QAMH), and Four (10% SPS in QAMH) for 10 min. Thaw solutions were kept at room temperature, and samples were protected from light and agitated while in thaw solutions.

### Tissue dissociation, culture, and fixation

**Fig. 1:**
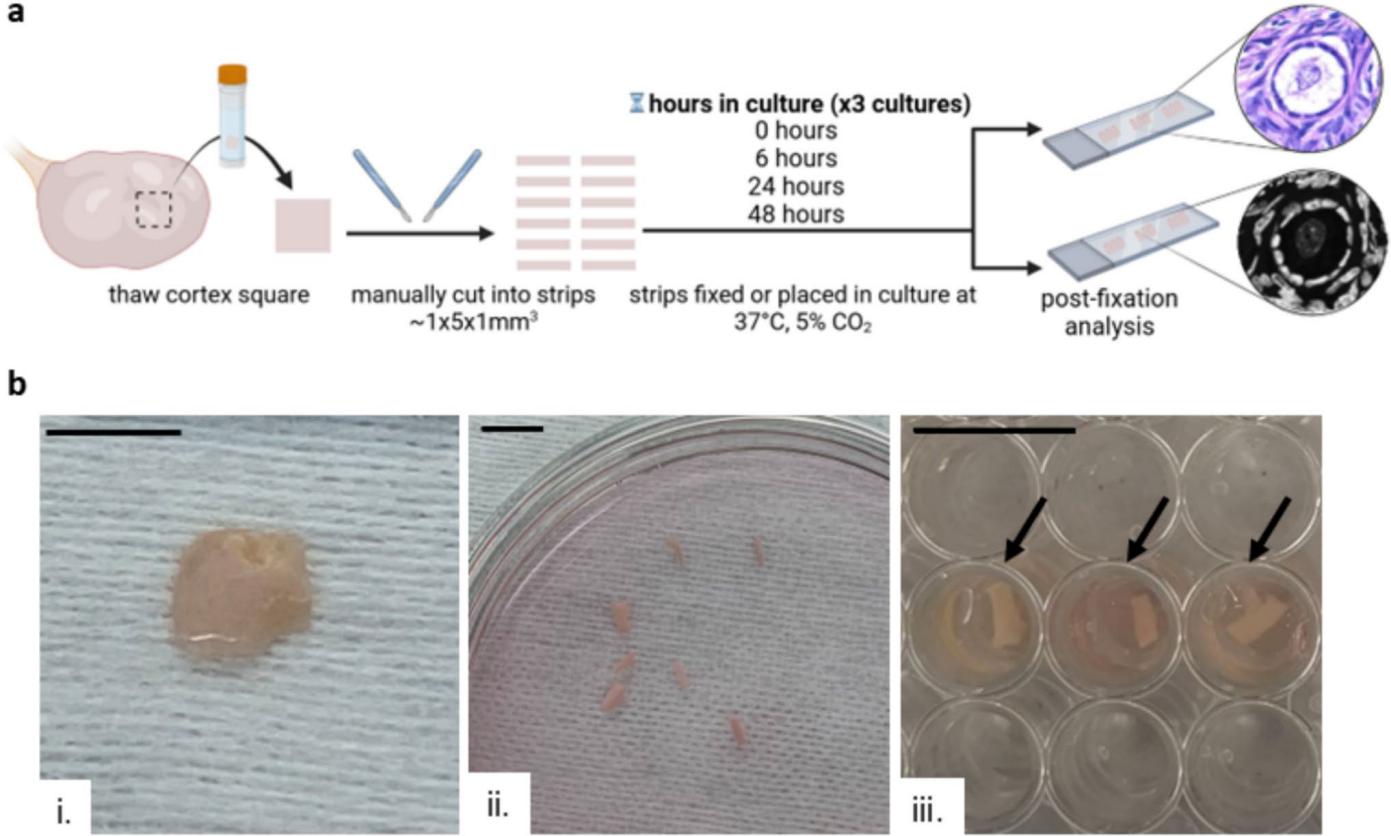
Visualization of experimental design. **a** Schematic representation of the experimental process. Frozen cortex squares were thawed and manually cut into strips ~ 5 mm × 1.5 mm × 1 mm^3^. Strips were fixed at 0 h post-fragmentation (T0) or placed in culture for 6 (T6), 24 (T24), or 48 (T48) h post-fragmentation. Post-fixation analysis included histological evaluation via H&E and fluorescent staining. The experiment was performed in triplicate. Created with BioRender.com. **b** Representative images of the ovarian cortex tissue throughout the experimental process show: (i) the cortex square after thawing, (ii) cortex strips after manual cutting, and (iii) three strips in wells of a culture plate prior to fixation, indicated by arrows. Scale bars: 1 cm

After thawing, the ovarian cortex square 10 mm × 10 mm × 1 mm was manually cut into approximately 12, ~ 5 mm × 1.5 mm × 1 mm strips at room temperature in Thaw Solution Four using a #10 disposal scalpel (Fischer Scientific, USA). Three strips were randomly selected as a Timepoint 0 (0 h post-fragmentation) sample and fixed in 4% paraformaldehyde (PFA) in PBS (Fisher Scientific) overnight at 4 °C, washed three times with Dulbecco’s Phosphate-Buffered Saline without calcium or magnesium (DPBS^−/−^) (Fisher Scientific), and stored in 70% ethanol for histological analysis. The remaining cortex strips were distributed randomly into three 96-well culture plates containing 150 µL growth media (GM) (α-MEM supplemented with 3 mg/mL human serum albumin (HSA, Fisher Scientific), 1 mg/mL bovine fetuin, 5 µg/mL insulin, 5 µg/mL transferrin, 5 ng/mL selenium (ITS, Sigma, St. Louis, MO, USA), and 10 mIU/mL highly purified, human-derived, follicle-stimulating hormone (FSH) (Urofollitropin, Ferring Pharmaceuticals, Saint-Prex, Switzerland) per well. The strips were incubated for 6 (Timepoint 6 (*n* = 3)), 24 (Timepoint 24 (*n* = 3)), and 48 h (Timepoint 48 (*n* = 3)). After culture, strips were fixed in 4% PFA in PBS (Fisher Scientific) overnight at 4 °C, washed three times with DPBS^−/−^ (Fisher Scientific), and stored in 70% ethanol. This experiment was performed in triplicate using Donor 1. The full experimental overview with representative images is summarized in Fig. 1.

### Histological analysis

Fixed tissue was embedded in paraffin blocks at the Histology Core in the Dental School at the University of Michigan, serially sectioned at a thickness of 5 µm with three sections per slide through the entirety of the tissue strips. Every other slide was stained with hematoxylin and eosin (H&E). Follicles in one of the three sections from each slide were counted and staged using brightfield microscopy (DM1000, Leica, Wetzlar, Germany). This equated to a volume between counted sections to 30 µm. All primordial and transitioning primordial follicles were counted for each of the slides. All primary and secondary follicles were only counted where the nucleus was visible to avoid “double-counting.” Follicles were staged based on standard morphological nomenclature previously described, and granulosa cells were defined as cuboidal if the ratio of length to width was between 0.5 and 2.0 [22]. Every follicle counted and staged was also annotated as morphologically normal or abnormal based on criteria previously specified, where no vacuoles or degeneration was seen in the oocyte or granulosa cells [23, 24]. Only follicles that were morphologically normal were quantified for further analysis. To obtain a percent stage distribution measurement for each timepoint, the total follicle count at each stage was divided by the total number of follicles counted in that timepoint and plotted using GraphPad Prism software. The percent of morphologically normal follicles out of total follicles was reported for the three experimental replicates across timepoints, within each follicle stage, and plotted using GraphPad Prism software.

### Immunofluorescence for the detection of YAP

**Fig. 2:**
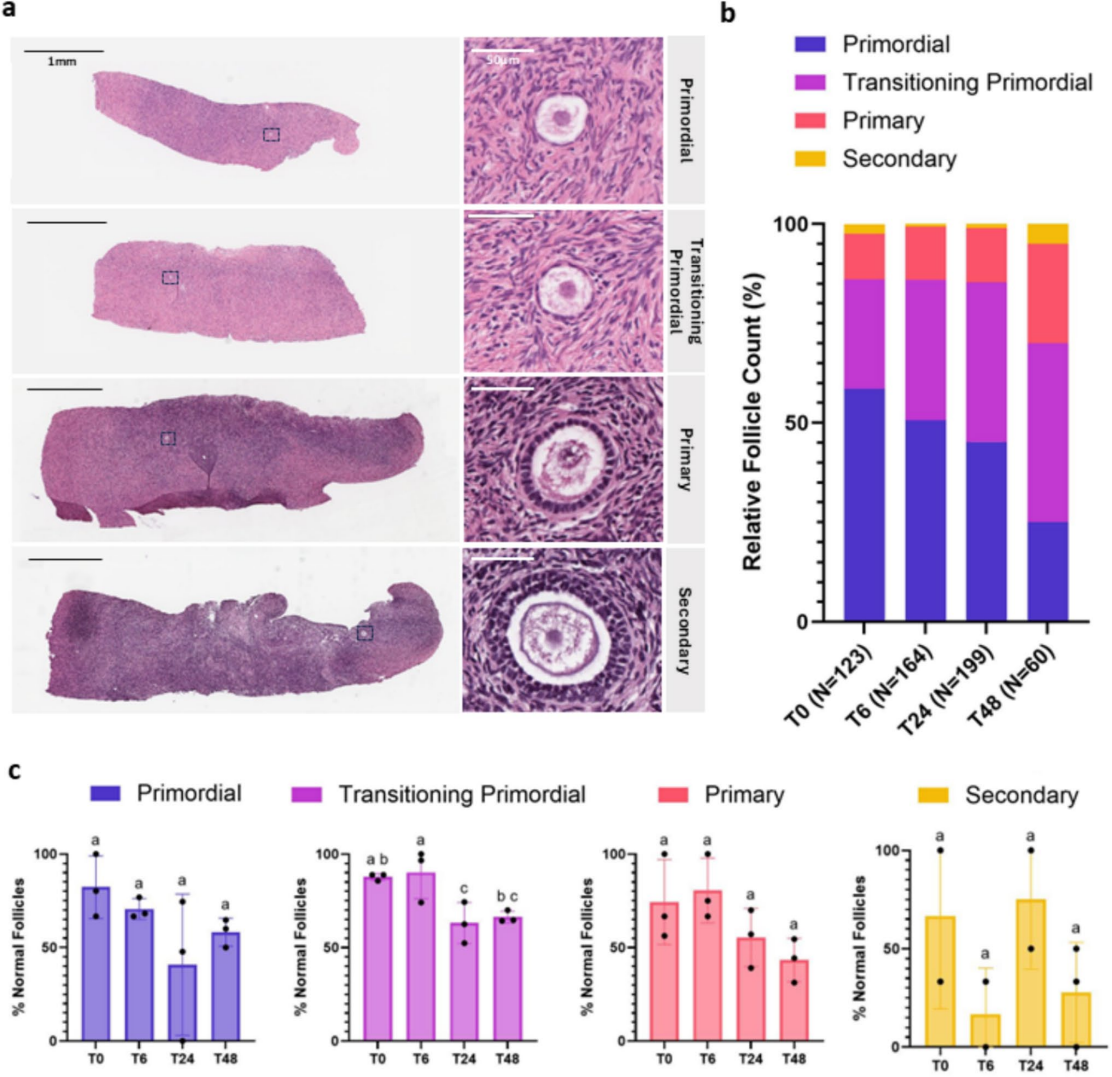
Histological analysis of ovarian cortex tissue from three replicates. **a** Representative histological images of ovarian cortex tissue strips containing each stage of folliculogenesis analyzed (primordial, transitioning primordial, primary, and secondary). Strip scale bars: 1 mm, callout scale bars: 50 µm. **b** Morphologically normal follicle stage distribution of three replicates across the four experimental timepoints, T0 h, T6 h, T24 h, and T48 h. The number of follicles measured at each timepoint is within the parentheses on the *x*-axis. **c** Plot showing the percentage of morphologically normal follicles analyzed across timepoints for primordial, transitioning primordial, primary, and secondary stages. Statistical significance was determined using an ordinary one-way ANOVA with Tukey’s multiple comparisons test, with *p* < 0.05 considered significant. Lowercase letters represent statistically significant differences, where groups that share a letter are not statistically significantly different, and those that do not share a letter are statistically significantly different

**Fig. 3:**
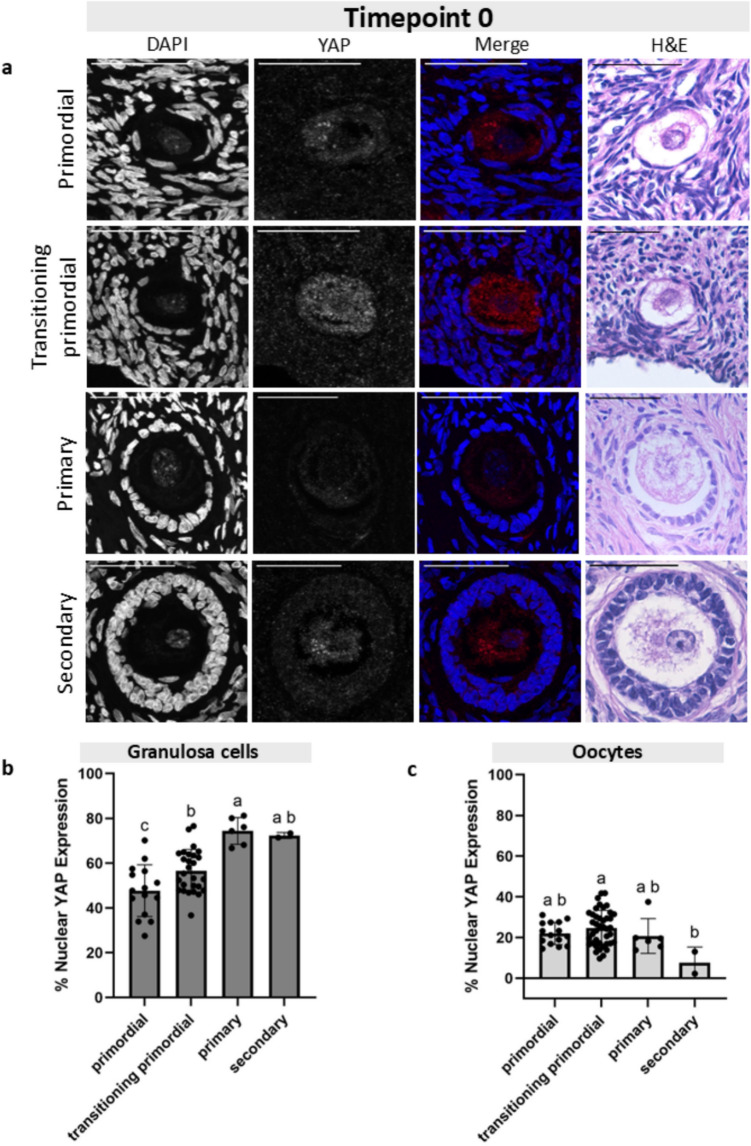
Granulosa cell and oocyte nuclear YAP expression at Timepoint 0 across follicle stages. **a** Representative images of primordial, transitioning primordial, primary, and secondary follicles from Timepoint 0 showing the DAPI channel, YAP channel, the merged image, and the corresponding H&E histological image. Scale bar: 50 µm. **b** The percentage of YAP that is nuclear within granulosa across four follicle stages shows a statistically significant increase in YAP nuclear expression from primordial to primary stages. **c** The oocyte percent nuclear YAP expression across four follicle stages shows little change in nuclear localization. Statistical significance was determined using an ordinary one-way ANOVA with Tukey’s multiple comparisons test, with *p* < 0.05 considered significant. Lowercase letters represent statistically significant differences, where groups that share a letter are not statistically significantly different, and those that do not share a letter are statistically significantly different

**Fig. 4:**
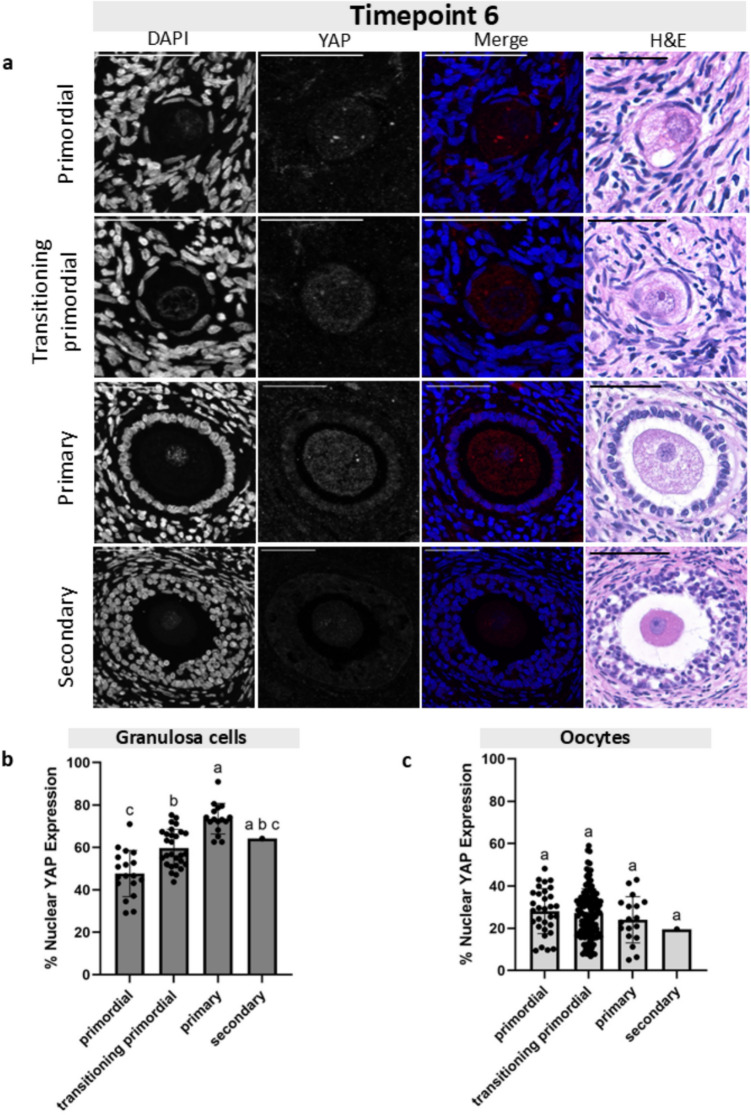
Granulosa cell and oocyte nuclear YAP expression at Timepoint 6 across follicle stages. **a** Representative images of primordial, transitioning primordial, primary, and secondary follicles from Timepoint 6 showing the DAPI channel, YAP channel, the merged image, and the corresponding H&E histological image. Scale bar: 50 µm. **b** Graph plotting the granulosa cell percent nuclear YAP expression across four follicle stages shows a statistically significant increase in YAP nuclear expression from primordial to primary stages. **c** Graph plotting the oocyte percent nuclear YAP expression across four follicle stages shows no statistically significant changes in nuclear localization. Statistical significance was determined using an ordinary one-way ANOVA with Tukey’s multiple comparisons test, with *p* < 0.05 considered significant. Lowercase letters represent statistically significant differences, where groups that share a letter are not statistically significantly different, and those that do not share a letter are statistically significantly different

**Fig. 5:**
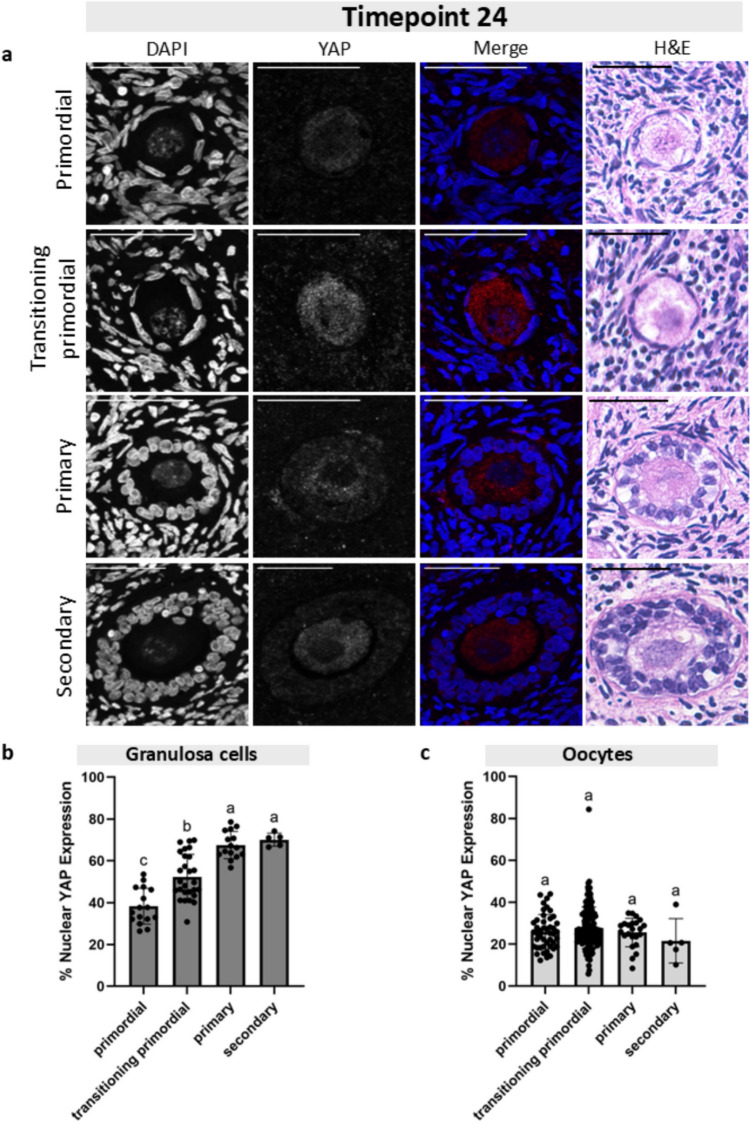
Granulosa cell and oocyte nuclear YAP expression at Timepoint 24 across follicle stages. **a** Representative images of primordial, transitioning primordial, primary, and secondary follicles from Timepoint 24 showing the DAPI channel, YAP channel, the merged image, and the corresponding H&E histological image. Scale bar: 50 µm. **b** Graph plotting the granulosa cell percent nuclear YAP expression across four follicle stages shows a statistically significant increase in YAP nuclear expression from primordial to primary stages. **c** Graph plotting the oocyte percent nuclear YAP expression across four follicle stages shows no statistically significant changes in nuclear localization. Statistical significance was determined using an ordinary one-way ANOVA with Tukey’s multiple comparisons test, with *p* < 0.05 considered significant. Lowercase letters represent statistically significant differences, where groups that share a letter are not statistically significantly different, and those that do not share a letter are statistically significantly different

**Fig. 6:**
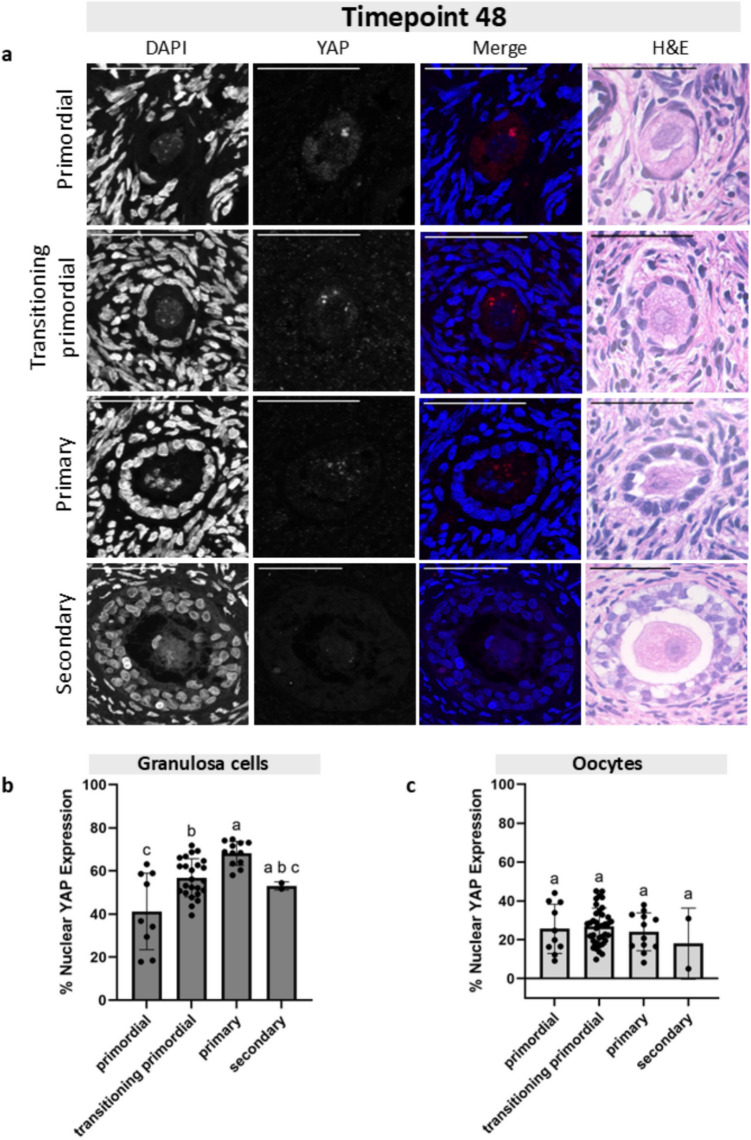
Granulosa cell and oocyte nuclear YAP expression at Timepoint 48 across follicle stages. **a** Representative images of primordial, transitioning primordial, primary, and secondary follicles from Timepoint 48 showing the DAPI channel, YAP channel, the merged image, and the corresponding H&E histological image. Scale bar: 50 µm. **b** Graph plotting the granulosa cell percent nuclear YAP expression across four follicle stages shows a statistically significant increase in YAP nuclear expression from primordial to primary stages. **c** Graph plotting the oocyte percent nuclear YAP expression across four follicle stages shows no statistically significant changes in nuclear localization. Statistical significance was determined using an ordinary one-way ANOVA with Tukey’s multiple comparisons test, with *p* < 0.05 considered significant. Lowercase letters represent statistically significant differences, where groups that share a letter are not statistically significantly different, and those that do not share a letter are statistically significantly different

**Fig. 7:**
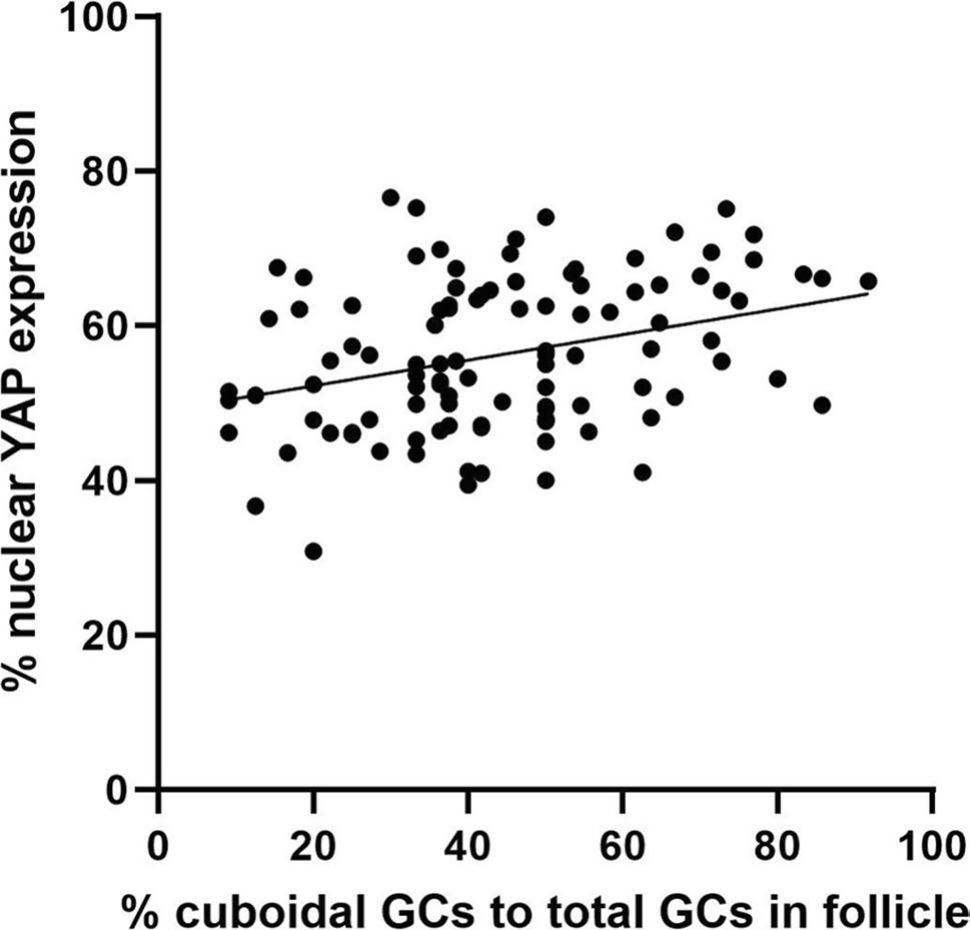
Correlation between an increased percentage of cuboidal granulosa cells and increasing granulosa cell layer nuclear YAP expression in transitioning primordial follicles. Transitioning primordial follicles from all timepoints (T0, T6, T24, and T48) were analyzed and combined. Graph plotting the percent cuboidal granulosa cells to total granulosa cells in each follicle compared to the percent nuclear YAP expression in the granulosa cell layer shows a positive correlation. A simple linear regression was performed where *R*.^2^ = 0.11, *p* = 0.00, and slope = 0.17

Slides were incubated for 1 h in a 60 °C dry oven the night before staining, deparaffinized in xylenes (Fisher Scientific), and rehydrated in decreasing concentrations of ethanol before undergoing blocking in 3% hydrogen peroxide diluted in MilliQ H_2_O for 30 min at room temperature. Slides then underwent heat-mediated antigen retrieval in 0.1 M sodium citrate buffer, pH 6 (Fisher Scientific), warmed to ~ 90 °C for 20 min, and allowed to cool for 20 min. Tissue was permeabilized in 0.1% Triton X-100 (Sigma Aldrich) in DPBS^−/−^ for 15 min at room temperature, followed by blocking for 1 h at room temperature in 500 mM glycine (BioRad, California, USA) and 10% normal goat serum (Fisher Scientific) in DPBS^−/−^. The primary antibody for YAP (sc-101199, Santa Cruz Biotechnology, Texas, USA) was diluted 1:50 in blocking solution, and slides were incubated with YAP antibody overnight at 4 °C. Samples were washed in 0.05% Tween-20 (Millipore Sigma, Massachusetts, USA) and 0.1% BSA (Fisher Scientific) in DPBS^−/−^ (3 ×, 20 min each) and then incubated with DAPI nuclear stain (Fisher Scientific) and Alexa Fluor 647 secondary antibody (Fisher Scientific) both diluted 1:1000 in blocking solution for 2 h at room temperature. Samples were washed in DPBS^−/−^ (3 ×, 20 min each) and then mounted on microscope slides with Prolong Diamond (Fisher Scientific). Experiments for YAP localization and quantification were performed using Donor 1 (Figs. 1, 2, 3, 4, 5, 6, and 7, Supplemental Figs. 2, 3, and 4).

### RNA fluorescence in situ hybridization (FISH) for the detection of CCN2

**Fig. 8:**
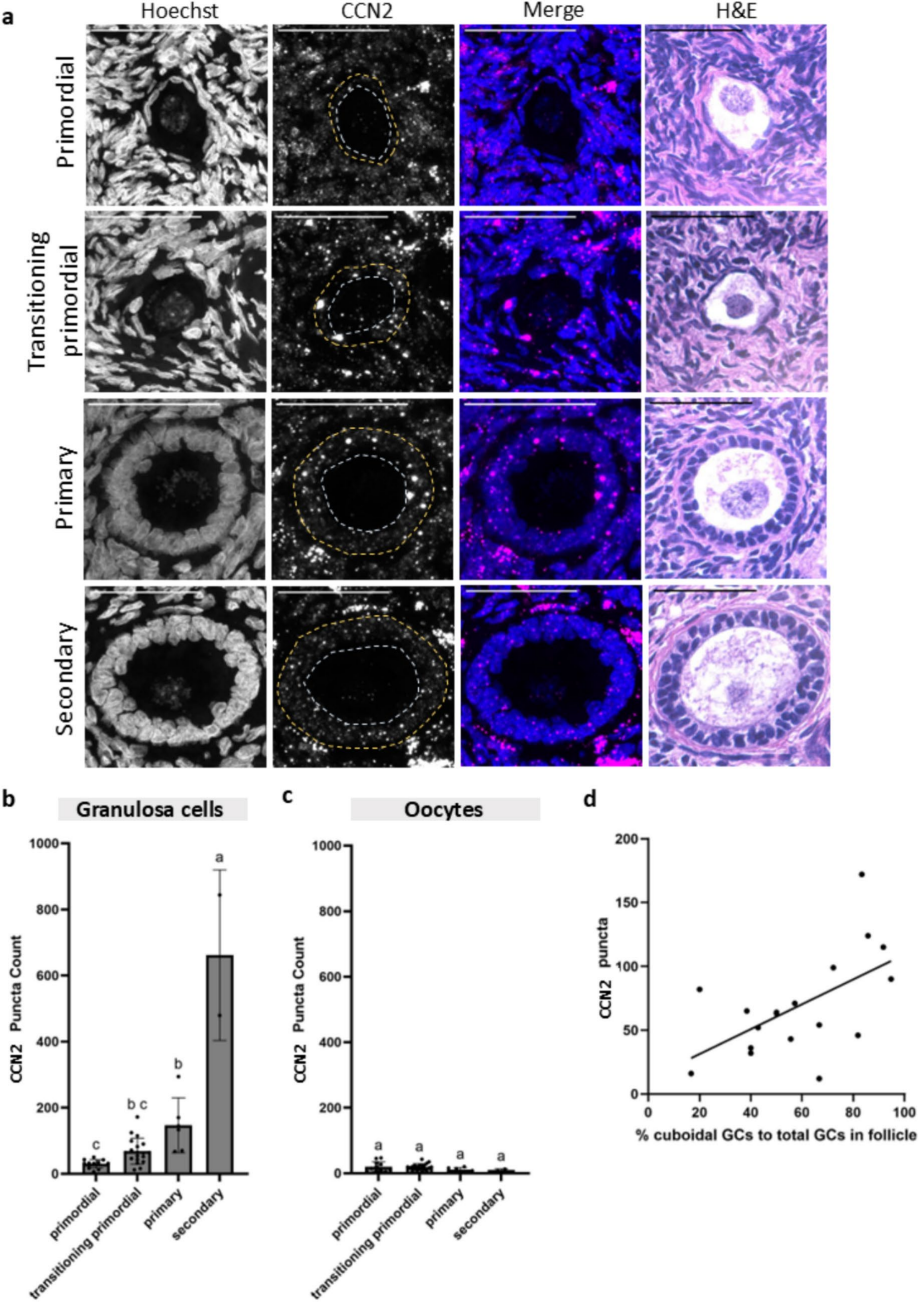
Granulosa cell and oocyte CCN2 mRNA expression at Timepoint 0 across follicle stages. **a** Representative images of primordial, transitioning primordial, primary, and secondary follicles showing Hoechst, CCN2, the merged image, and the corresponding H&E histological image. Scale bar: 50 µm. Yellow dashes indicate the follicle boundary, while light blue dashes indicate the oocyte boundary. **b** Granulosa cell CCN2 mRNA puncta counts across four follicle stages show a statistically significant increase in CCN2 expression from primordial to primary stages. **c** Oocyte CCN2 mRNA puncta counts across four follicle stages show no statistically significant changes across stages. Statistical significance was determined using an ordinary one-way ANOVA with Tukey’s multiple comparisons test, with *p* < 0.05 considered significant. Lowercase letters represent statistically significant differences, where groups that share a letter are not statistically significantly different, and those that do not share a letter are statistically significantly different at a *p* value of at least 0.05. **d** Graph plotting the percent cuboidal granulosa cells to total granulosa cells in each transitioning primordial follicle compared to CCN2 mRNA puncta counts in the granulosa cell layer shows a positive correlation. A simple linear regression was performed where *R*.^2^ = 0.032, *p* = 0.01, and slope = 0.98

Slides were stored at room temperature in a slide box containing a silica desiccator packet prior to staining. Slides were incubated for 1 h in a 60 °C dry oven the night before staining. RNA FISH probes were acquired from Advanced Cell Diagnostics (ACD) and stained using the RNAscope multiplex fluorescent manual protocol and kit (RNAscope Multiplex Fluorescent Reagent Kit v2, ACD, Newark, CA). The RNAscope Probe Hs-CTGF (ACD Catalog 560,581) was used. Note that CTGF and CCN2 refer to the same gene, and we use the gene name CCN2 throughout the manuscript. The RNA FISH protocol was performed according to the manufacturer’s instructions (ACD document number 323100-USM) with a 15-min antigen retrieval and a 45-min protease treatment. Slides were counterstained with Hoechst 33,342 (Fisher Scientific) for 30 min at room temperature and mounted on microscope slides with Prolong Diamond (Fisher Scientific). Experiments for CCN2 quantification were performed using Donor 2 (Fig. 8).

### Confocal microscopy and quantification of YAP nuclear expression and CCN2 expression

Images were captured on a Leica SP8 laser scanning confocal microscope. All confocal images were captured using the same laser power, gain, and imaging parameters for each stain. Follicles were imaged at the cross-section where the oocyte nucleus was clearly visible. All YAP nuclear quantification and CCN2 quantification were performed in FIJI [25] on unaltered images that contained metadata. Methods for quantifying the percent nuclear YAP expression in oocytes and granulosa cells are outlined in Fig. S1. Briefly, to calculate the percent nuclear YAP expression in oocytes, an ROI was created around the oocyte nucleus (1) and the entire oocyte (2). These ROIs were overlaid onto the YAP channel, and the integrated density was measured. Then, the percent nuclear YAP expression was determined by dividing the integrated density of the entire oocyte (2) by the oocyte nucleus (1) and multiplying by 100. To calculate the percent nuclear YAP expression in the granulosa cells, an ROI was created around the whole follicle (3) and around each individual granulosa cell nucleus (4). These ROIs were then overlaid onto the YAP channel to measure integrated density. To calculate the granulosa cell layer YAP expression, the oocyte integrated density (3) was subtracted from the entire follicle integrated density (4). Finally, the percent nuclear YAP expression was calculated by dividing the integrated density of all the granulosa cell nuclei (4) by the entire granulosa cell layer and multiplying by 100. For transitioning primordial follicles only, each individual granulosa cell measured was also classified as squamous or cuboidal based on morphology. To quantify the expression of CCN2 in follicles, an ROI was created around the follicle and the oocyte at the center z-stack slice for each image. Images were then subjected to thresholding and watershed. Individual mRNA puncta were counted using the “analyze particles” feature in FIJI. The individual mRNA puncta were reported for the granulosa cell layer (follicle minus oocyte) and for the oocyte. A complete list of the number of slides and follicles analyzed for YAP and CCN2 mRNA puncta quantification is summarized in Table S1. The granulosa cell YAP quantification measurements were performed on the same pool of follicles as the oocyte quantification measurements. Images of positive YAP staining compared to two follicles with secondary antibodies only can be seen in Fig. S2.

### Statistical analysis

Statistical analysis was performed using GraphPad Prism 10. All graphs showing percent nuclear YAP expression across follicle stages and timepoints and graphs showing CCN2 integrated density were plotted as mean ± SD, and statistical significance was determined using an ordinary one-way ANOVA with Tukey’s multiple comparisons test (*p* < 0.05). Different lowercase letters represent statistically significant differences with a *p* value of at least 0.05, while groups that share the same letter are not statistically significantly different. Graphs correlating the percent cuboidal granulosa cells to total granulosa cells in the follicle with the percent nuclear YAP expression used a simple linear regression with R^2^, *p* value, and slope reported.

## Results

### Follicles at four distinct stages of folliculogenesis were present within human ovarian cortex tissue strips across four experimental timepoints

The histological analysis of human ovarian cortex strips at each timepoint revealed follicles ranging from primordial, transitioning primordial, primary, and secondary follicle stages (Fig. 2a). We analyzed a total of 546 morphologically normal follicles across four timepoints and three experimental replicates and determined how the follicle distributions shifted with longer time in culture (Fig. 2b and Table 2). At Timepoint 0 (T0), immediately after ovarian tissue fragmentation, we detected 123 follicles in 103 sections with 58.5% primordial, 27.6% transitioning primordial, 11.4% primary, and 2.4% secondary. After 6 h of culture (T6), the follicle distribution of a total of 164 follicles from 138 sections was similar to T0, with 50.6% primordial, 35.4% transitioning primordial, 13.4% primary, and 0.6% secondary. After 24 h in culture (T24), the distribution of follicles shifted toward more advanced stages. From the 199 follicles in 188 sections analyzed at T24, 45.2% were primordial, 40.2% transitioning primordial, 13.6% primary, and 1% secondary. Finally, after 48 h of culture (T48), the distribution of a total of 60 follicles was 25.0% primordial, 45.0% transitioning primordial, 25.0% primary, and 5.0% secondary. Interestingly, the number of primordial follicles decreased, with 34% primordial follicles after 48 h in culture, and the percentage of growing follicles increased, with 17% for transitioning primordial follicles and 14% for primary follicles, suggesting follicle activation and recruitment to the growing pool. This decrease in primordial follicles, followed by a subsequent increase in transitioning and primary follicle stages during a short-term tissue culture, was also seen in another study that reported a 20% decrease in primordial follicle distribution from their culture day 0 to culture day 2 and an increase in later follicle stages after 2 days [15].

**Table 2:** Normal histological follicle count summary

Follicle counts (%)					Total follicle count	Total volume counted (mm^3^)
Timepoint	Primordial	Transitional primordial	Primary	Secondary		
T0	72 (58.5)	34 (27.6)	14 (11.4)	3 (2.4)	123	76.0
T6	83 (50.6)	58 (35.4)	22 (13.4)	1 (0.6)	164	117.7
T24	90 (45.2)	80 (40.2)	27 (13.6)	2 (1.0)	199	83.0
T48	15 (25.0)	27 (45.0)	15 (25.0)	3 (5.0)	60	105.8

Table expands upon Fig. 2b, detailing the number of follicles counted at each stage of folliculogenesis, followed by the percent stage distribution out of the total follicle count for all four timepoints.

To determine if the shifts in follicle distribution between timepoints were due to follicle atresia, as opposed to increased growth, we plotted the percentage of morphologically normal follicles from the three replicates across timepoints at each follicle stage (Fig. 2c). There were no statistically significant differences between the number of healthy primordial follicles across timepoints, suggesting that the primordial follicle proportion is not decreasing due to follicle death. Furthermore, it was encouraging to see that the increase in the proportion of later stages of follicles seen in T24 and T48 was not due to there being more unhealthy follicles, as both the primary and secondary stages showed no statistically significant differences in follicle morphology at these later timepoints.

### Nuclear localization of YAP in granulosa cells and oocytes remained consistent across time in culture within each follicle stage

The decrease in primordial follicle number followed by an increase in later-stage follicles at T48 indicated potential follicle activation across culture. Therefore, we investigated whether YAP nuclear localization changes in each class of follicles over time in culture. We plotted the percent nuclear YAP expression across timepoints for each follicle stage for both GCs and oocytes (Fig. S3). Nuclear YAP expression remained constant across timepoints at each follicle stage but was always higher in granulosa cells compared to oocytes. In primordial follicles, GC nuclear YAP expression across timepoints was 43.7%, while oocyte nuclear expression averaged at 25.4%. In transitioning primordial follicles, GC average nuclear YAP was 56.3%, while oocytes averaged at 26.6%. Primary follicles contained an average nuclear GC YAP expression of 70.9% and oocyte expression of 23.6%. Secondary follicles contained an average nuclear expression of 65.0% and 16.7% for GCs and oocytes, respectively. Overall, our results show that nuclear YAP expression is overall higher in granulosa cells compared to oocytes at primordial, transitioning primordial, primary, and secondary stages, and its expression levels do not change during follicle culture.

### YAP accumulated in the nuclei of granulosa cells but not oocytes across folliculogenesis

After we established that the percent nuclear YAP expression was the same in follicles at the same stage across the four culture timepoints, we analyzed follicles at different development stages within each culture timepoint. We quantified the proportion of YAP expression in the nucleus of GCs and oocytes in primordial, transitioning primordial, primary, and secondary follicles at four timepoints of the tissue culture (T0, T6, T24, and T48) (Figs. 3, 4, 5, and 6). At T0, the average nuclear YAP expression in GCs of the 15 primordial follicles analyzed was 47.7% (Fig. 3b). The proportion of nuclear YAP expression in transitioning primordial follicles was 56.6% and was significantly greater in the 27 follicles analyzed. The proportion of nuclear YAP expression in primary follicles was also significantly greater, with an average of 74.4%. There was no difference in nuclear YAP expression in GCs between the primary and secondary stage follicles (72.4% nuclear GC YAP), but overall, the nuclear proportion of YAP at the secondary stage showed significantly higher abundance than the primordial stage. To analyze YAP localization in oocytes, we measured 15 primordial, 41 transitioning primordial, 6 primary, and 2 secondary stage follicles. In oocytes, YAP showed little change in the nuclear proportion across all four follicle stages, and the mean expression remained quite low, below 40% (Fig. 3c). A similar pattern of the proportion of nuclear YAP expression in both GCs and oocyte was observed at T6 (Fig. 4), T24 (Fig. 5), and T48 (Fig. 6). At all three of these timepoints, the proportion of nuclear YAP expression in GCs showed statistically significant greater abundance as follicles progressed from the primordial to primary stages and leveled off at the secondary stage, whereas the proportion of YAP in the oocyte nuclei showed no statistically significant differences across follicle stages, always averaging below 40%.

### Transitioning primordial follicles containing a greater proportion of cuboidal granulosa cells out of the total number of granulosa cells showed greater nuclear YAP localization

During primordial follicle activation, the squamous, quiescent GCs surrounding the oocyte transition to a cuboidal morphology, which is often followed by proliferation [26, 27]. These transitioning primordial follicles are uniquely distinguishable due to the presence of a mixed population of both cuboidal and squamous GCs. YAP is known to transcribe pro-proliferative and anti-apoptotic genes and is proposed to be involved with follicle activation in mice. We hypothesized that transitioning primordial follicles with a higher proportion of cuboidal GCs would also have a higher percent of overall nuclear YAP signal. We tested this by analyzing the morphology and nuclear YAP expression of 1125 individual GCs from 106 transitioning primordial follicles (Table S1). Based on our observation that YAP nuclear accumulation is timepoint-independent (Fig. S3), we combined transitioning primordial follicles across all four timepoints in our analysis. We found that the percent nuclear YAP expression in the GC layer increases with an increasing proportion of cuboidal GCs within follicles (*R*^2^ = 0.11, *p* = 0.0007, and slope = 0.17, Fig. 7). Figure S4 shows this correlation breakdown by individual timepoint with a similar positive correlation. This finding suggests that follicles transitioning from dormancy to activation accumulate YAP in the nucleus of GCs, but not the oocytes.

### Connective tissue growth factor (CCN2) mRNA expression increases in granulosa cells as follicles transition from the primordial to the secondary stage of folliculogenesis

One of the key target genes induced by YAP is connective tissue growth factor (CTGF), also known as cellular communication network factor 2 (CCN2) [28, 29]. To test whether our observed increase in YAP nuclear localization in GCs from primordial to primary follicles translated to an increase in YAP transcriptional activity, we measured the expression of CCN2 mRNA in GCs and oocytes across primordial, transitioning primordial, primary, and secondary stages at T0 (Fig. 8, Table S1). There were significantly more transcripts of CCN2 mRNA in granulosa cells of primary follicles (mean mRNA puncta = 146.5) than in primordial follicles (mean mRNA puncta = 30.0) (Fig. 8b). Consistent with the YAP expression pattern, CCN2 expression in oocytes was low and not statistically significantly different between any of the stages (mean mRNA puncta ≤ 20.09, Fig. 8c). Previously, we saw that YAP nuclear expression increased in transitioning primordial follicles containing a higher proportion of activated cuboidal GCs. We tested whether the increased expression of nuclear YAP reflected its transcriptional activity by analyzing 206 GCs from 19 transitioning primordial follicles and determined the proportion of cuboidal GCs to total GCs as well as the total granulosa cell layer CCN2 mRNA expression (Fig. 8d, Table S1). This analysis showed a positive correlation between increased cuboidal GCs in a follicle and an increase in CCN2 mRNA puncta (*R*^2^ = 0.32, *p* = 0.01, and slope = 0.98, Fig. 8d). Overall, CCN2 expression in GCs followed the same pattern as YAP expression. CCN2 mRNA significantly increased from the primordial to primary stages, remained unchanged in oocytes, and increased as follicles transitioned through activation, suggesting increased YAP transcriptional activity in activated follicles.

## Discussion

This study tested whether Hippo pathway disruption is a driving mechanism of follicle activation in human ovarian cortex tissue by examining YAP nuclear localization and expression of a key YAP target gene, CCN2, in granulosa cells and oocytes across four distinct follicle stages from four timepoints post ovarian tissue fragmentation and culture. We report for the first time a consistent and statistically significant increase in nuclear YAP abundance in granulosa cells from the primordial to primary stage of folliculogenesis. Furthermore, the increased nuclear YAP expression is reflected in a subsequent statistically significant increase in CCN2 mRNA transcripts in granulosa cells. CCN2 has been shown to be upregulated by mechanical stress and modulates several growth factors that stimulate cell growth, survival, and proliferation [30–32]. Together, these data suggest that YAP acts within transitioning and later-stage follicles—at the level of the granulosa cells—beyond the primordial follicle stage and remains transcriptionally active as follicles grow.

Studies in mice have shown that knocking down YAP in ovaries led to inhibition of follicle growth, where the majority of follicles remained in the primordial follicle stage, while overexpression of YAP resulted in an increased population of growing follicles [9, 33, 34]. Additionally, it was found that YAP was localized in the nucleus of granulosa cells from growing follicles and that specific inactivation of YAP in granulosa cells within an in vitro culture system disrupted follicle development [34]. Consistent with these studies, we saw a distinct accumulation of YAP in human granulosa cells, as follicles transitioned from the primordial to primary stages. We did not observe nuclear accumulation of YAP in human oocytes, also consistent with previous findings in mice proposing multiple mechanisms that prevent YAP nuclear accumulation in oocytes at any stage of development [35]. Furthermore, studies demonstrated that fragmenting mouse ovaries led to the accumulation of YAP in the nucleus of granulosa cells and a subsequent increase in CCN transcripts [5, 10, 11]. We further confirmed that YAP is transcriptionally active in granulosa cells of growing follicles, which showed increased numbers of CCN2 transcripts. Together, these findings reinforce the critical and conserved role of YAP activity during follicle activation in humans.

Limited prior research on the Hippo pathway in human ovarian cortex tissue has yielded mixed results, highlighting the need for this investigation. One study examined the Hippo pathway during in vitro follicle culture over time, comparing ovarian tissue derived from oncological patients and transgender men undergoing ovariectomy [15]. Although this study did not quantify the YAP immunofluorescent signal, it reported that YAP was expressed in granulosa cells of primordial, transitioning primordial, primary, and secondary follicles throughout the culture period, which is consistent with our findings. This group also noted a decrease in YAP expression in oocytes from primordial to later follicle stages on day 0, but not on subsequent culture days. We found no change in YAP expression in oocytes across follicle stages for any of our experimental timepoints and similarly saw low transcriptional output of CCN2 in oocytes; however, these conflicting findings may be due to differences in tissue manipulation. In that specific study, the ovarian cortex tissue was pulled mechanically to flatten out the tissue after fragmentation, which may have further altered Hippo pathway mechanics. Another group reported a statistically significant increase in YAP nuclear expression in human primordial follicle granulosa cells 3days after grafting the ovarian tissue into SCID mice [17]. Though we did not see any changes in YAP nuclear localization in primordial follicle granulosa cells up to 48 h, it is possible that fragmentation-induced effects do not manifest until later at this stage. Furthermore, in vitro culture conditions may impact fragmentation-induced YAP dynamics differently than in vivo grafts, a hypothesis that requires investigation through longer-term cultures.

Our findings provide a novel angle to the fact that YAP activity in granulosa cells and not the oocyte could be the key regulator of human follicle activation, a role that is likely conserved between humans and mice. However, our study also has a few limitations. The histological follicle staging methods in our study relied on analyzing granulosa cell morphology on the same plane as the largest oocyte cross-section, where the nucleus was visible, potentially missing the granulosa cells with alternate morphology in different planes and leading to follicle stage misclassification. Additionally, Hippo signaling and YAP dynamics may depend on culture conditions and whether the tissue is fresh or previously cryopreserved and thawed. Future research looking at YAP dynamics in fresh versus frozen/thawed tissue conditions would be important for clinical applications. Finally, due to the limited availability of healthy reproductive-age human ovarian tissue donations, we analyzed tissue from only two donors in this study, who were overweight and obese, an important confounding factor in this study. Increasing the number of donors and the variety of ages and BMIs would be important to confirm and validate the conclusions of our study.

This research opens several avenues for future investigations. For example, the dynamics of Hippo signaling in follicle activation could change drastically in aging ovaries that are stiffer and more fibrotic, which may influence mechanosensing [36]. Additionally, we noticed an increased presence of CCN2 mRNA puncta in theca cells surrounding secondary follicles (Fig. 8a), suggesting a potentially novel role of the Hippo pathway within the newly formed theca layer from the secondary stage of development and onwards. Furthermore, to understand the role of the Hippo pathway in human follicle activation, interventions to induce or prevent follicle activation could be tested. For example, treating mouse ovaries with actin-polymerization-enhancing agents such as Jasplakinolide (JASP) and sphingosine-1-phosphate (S1P) promoted YAP nuclear localization in follicles and increased CCN2 transcripts [37]. Applying this or other strategies to human ovarian tissue would provide critical functional evidence for the role of YAP in human follicle activation. Ultimately, understanding the mechanisms behind follicle activation is crucial for regulating this process in vitro, paving the way for future advancements in fertility preservation.

## Supplementary Information

Below is the link to the electronic supplementary material.

ESM 1 (PDF 560 KB)

## Data Availability

Not applicable.
