# Supplementary material for: Dynamics of YAP localization and transcript activity in human oocytes and granulosa cells across early-stage folliculogenesis: an exploratory investigation
Source: J Assist Reprod Genet. 2025 Sep 26;42(12):4221–36. doi: 10.1007/s10815-025-03668-2 (PMC12705925; doi:10.1007/s10815-025-03668-2)
Supplement: Supplementary file 1 — (PDF 560 KB) [file 10815_2025_3668_MOESM1_ESM.pdf]

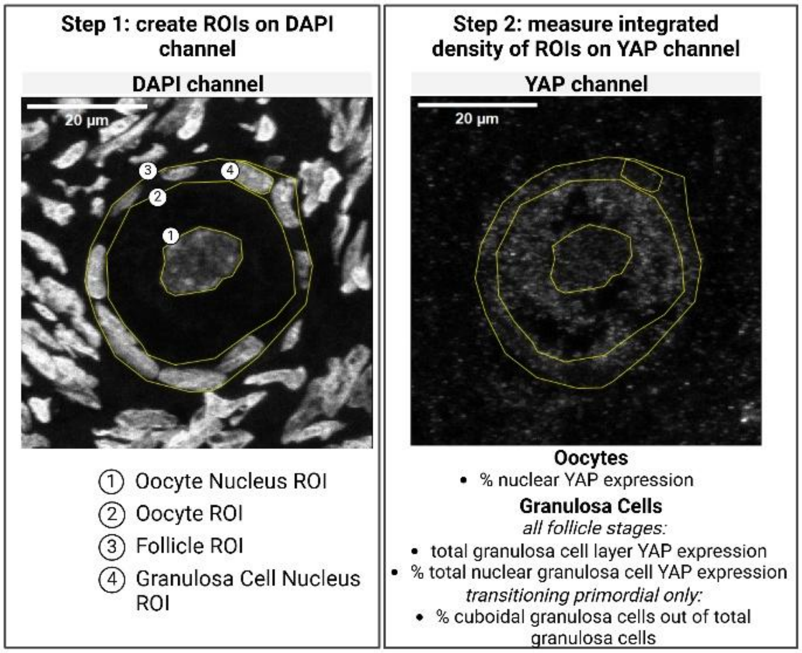

Supplementary Fig. S1 Method of quantifying nuclear YAP integrated density in oocytes and granulosa cells. In step 1 an ROI was created on the DAPI channel around (1) the oocyte nucleus (2) the oocyte (3) the follicle and (4) the granulosa cells. In step 2 the ROIs were overlayed on the YAP channel and the integrated density of each ROI is measured. The following measurements were obtained: the oocyte percent nuclear YAP expression, the total granulosa cell layer YAP expression, the percent total nuclear granulosa cell YAP expression, and the percent cuboidal granulosa cells out of the total granulosa cells (for transitioning primordial follicles only)

Supplemental Table 1. YAP and CCN2 Quantification Sample Overview

Oocyte YAP Quantification

	Number of slides stained	Number of follicles analyzed			
		primordial	transitioning primordial	primary	secondary
Timepoint 0	64	15	41	6	2
Timepoint 6	74	31	124	18	1
Timepoint 24	70	45	152	23	5
Timepoint 48	60	10	37	12	2
Total	268	101	354	59	10
		524			

Granulosa Cell YAP Quantification

	Number of slides stained	Number of follicles analyzed			
		primordial	transitioning primordial	primary	secondary
Timepoint 0	64	15	27 (298)*	6	2
Timepoint 6	74	18	28 (317)*	16	1
Timepoint 24	70	16	27 (248)*	15	5
Timepoint 48	60	9	24 (262)*	12	2
Total	268	58	106 (1125)*	49	10
		223			

CCN2 mRNA Quantification

	Number of slides stained	Number of follicles analyzed			
		primordial	transitioning primordial	primary	secondary
Timepoint 0	18	11	19 (206)*	6	2
	Total	38			

*number in parentheses indicates total number of granulosa cells analyzed

Supplementary Table S1. YAP and CCN2 quantification sample overview. The table details the exact number of slides and individual follicles measured to quantify YAP immunofluorescence and CCN2 mRNA puncta in both oocytes and granulosa cells across four timepoints. Each individual granulosa cell from transitioning primordial follicles was quantified and staged as squamous or cuboidal. The number of granulosa cells analyzed is indicated within the parenthesis.

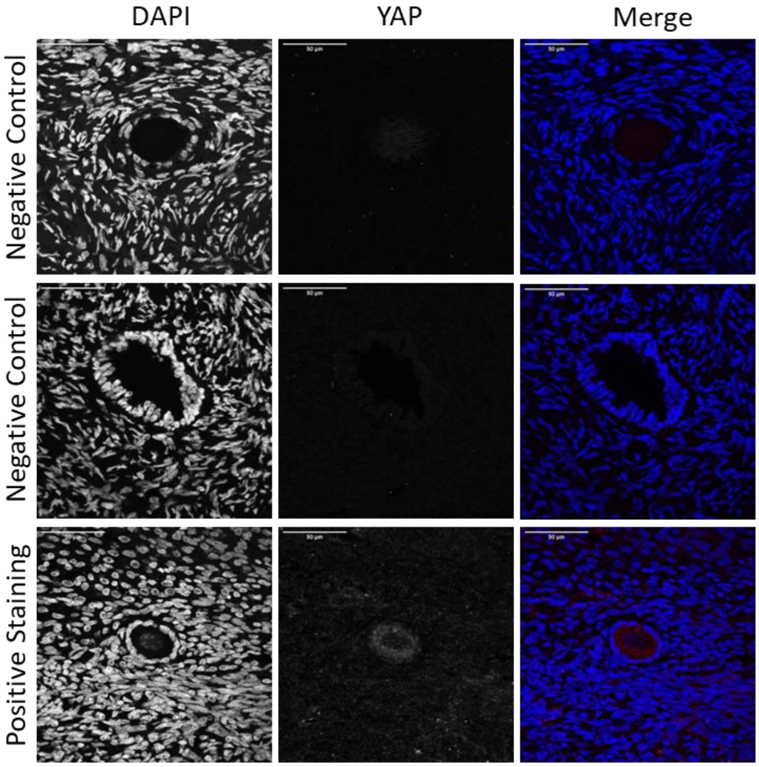

Supplementary Fig. S2 Representative images of YAP negative controls compared to positive staining. First two panels show representative images of secondary-only stain slides (negative controls) for YAP staining in an earlier stage (top panel) and a later stage (middle panel) follicle. Last panel shows an example of positive YAP staining for comparison. DAPI channel, YAP channel, and the merged image are shown. Scale bar: 50µm.

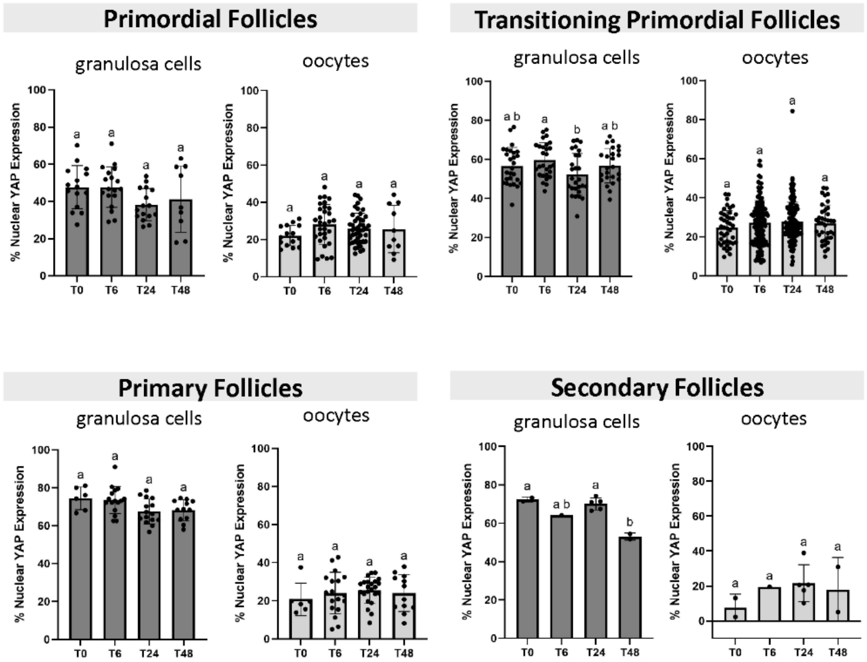

Supplementary Fig. S3 Granulosa cell and oocyte nuclear YAP expression across timepoints within each follicle stage. Granulosa cell and oocyte nuclear YAP expression within each follicle stage across four timepoints. Statistical significance was determined using an ordinary one-way ANOVA with Tukey's multiple comparisons test with $p < 0.05$ considered significant. Lowercase letters represent statistically significant differences, where groups that share a letter are not statistically significantly different and those that do not share a letter are statistically significantly different at a *p* value of at least 0.05

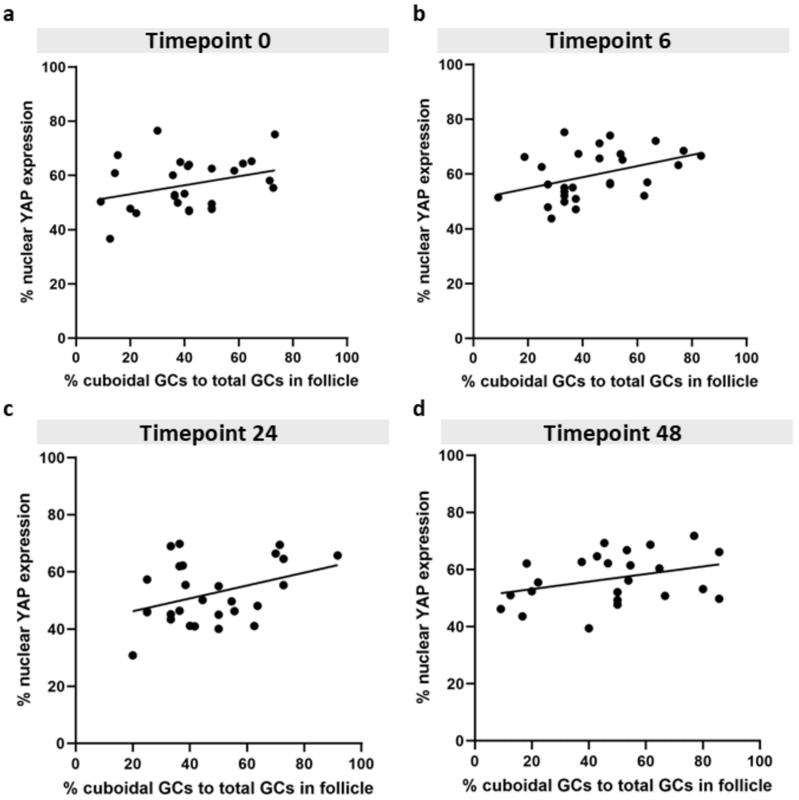

Supplementary Fig. S4 Correlation between an increased percentage of cuboidal granulosa cells and increasing granulosa cell layer nuclear YAP expression plotted across four timepoints. Transitioning primordial follicles were analyzed at each individual timepoint. **a.** Graph plotting the percent cuboidal granulosa cells to total granulosa cells in each follicle compared to the percent nuclear YAP expression in the granulosa cell layer at Timepoint 0. A simple linear regression was performed where $R^2 = 0.10$, $p = 0.11$, and slope = 0.16. **b.** Graph plotting the percent cuboidal granulosa cells to total granulosa cells in each follicle compared to the percent nuclear YAP expression in the granulosa cell layer at Timepoint 6. A simple linear regression was performed where $R^2 = 0.17$, $p = 0.03$, and slope: 0.20. **c.** Graph plotting the percent cuboidal granulosa cells to total granulosa cells in each follicle compared to the percent nuclear YAP expression in the granulosa cell layer at Timepoint 24. A simple linear regression was performed where $R^2 = 0.14$, $p = 0.05$, and slope = 0.23. **d.** Graph plotting the percent cuboidal granulosa cells to total granulosa cells in each follicle compared to the percent nuclear YAP expression in the granulosa cell layer at Timepoint 48. A simple linear regression was performed where $R^2 = 0.12$, p value = 0.10, and slope = 0.13
